# Technical Aspects of Laparoscopic Distal Pancreatectomy for Benign and Malignant Disease: Review of the Literature

**DOI:** 10.1155/2015/472906

**Published:** 2015-07-09

**Authors:** T. de Rooij, R. Sitarz, O. R. Busch, M. G. Besselink, M. Abu Hilal

**Affiliations:** ^1^Department of Surgery, Academic Medical Center, Meibergdreef 9, 1105 AZ Amsterdam, Netherlands; ^2^Department of Surgical Oncology, Medical University of Lublin, Lublin, Poland; ^3^Department of Surgery, Southampton University Hospital, Southampton, UK

## Abstract

Distal pancreatectomy is the standard curative treatment for symptomatic benign, premalignant, and malignant disease of the pancreatic body and tail. The most obvious benefits of a laparoscopic approach to distal pancreatectomy include earlier recovery and shorter hospital stay. Spleen-preserving distal pancreatectomy should be attempted in case of benign disease. Spleen preservation can be achieved preferably by preserving the splenic vessels (Kimura technique), but also by resecting the splenic vessels and maintaining vascularity through the short gastric vessels and left gastroepiploic artery (Warshaw technique). Several studies have suggested a higher rate of spleen preservation with laparoscopy. The radical antegrade modular pancreatosplenectomy has become mainstay for treating pancreatic cancer and can be performed laparoscopically as well. Evidence on the feasibility and safety of laparoscopic distal pancreatectomy for cancer is scarce. Despite the obvious advantages of laparoscopic surgery, postoperative morbidity remains relatively high, mainly because of the high incidence of pancreatic fistula. For decades, surgeons have tried to prevent these fistulas but to date no strategy has been confirmed to be effective in 2 consecutive randomized studies. Pragmatic multicenter studies focusing on technical aspects of laparoscopic distal pancreatectomy are lacking and should be encouraged.

## 1. Introduction

Distal pancreatectomy is the standard treatment for symptomatic benign, premalignant, and malignant lesions in the pancreatic body and tail. Distal pancreatectomy includes resection of pancreatic tissue to the left of the portomesenteric vein and can be extended with lymphadenectomy and splenectomy in case of malignant disease. Through the years, this procedure has been tailored to specific indications with the objective of improving postoperative outcomes and minimizing patient trauma. A laparoscopic approach to distal pancreatectomy has been suggested to be associated with reduced postoperative morbidity and hospital stay compared with open surgery [1, 2]. However, this approach is considered to be technically more demanding than conventional surgery.

In case of symptomatic benign or premalignant disease, attempts should be made to preserve the spleen, with either splenic vessel preservation (described by Kimura et al. [3]) or resection of the splenic vessels (described by Warshaw [4]), because a splenectomy is incapacitating for patients as it necessitates vaccinations and supply of antibiotics in order to prevent potentially lethal postsplenectomy sepsis. Also, in order to prevent overwhelming postsplenectomy sepsis, the use of antibiotic prophylaxis after splenectomy is advised by several guidelines worldwide [5].

Unfortunately, the rate of pancreatic fistula after distal pancreatectomy (POPF) remains high. Surgeons have tried to prevent these fistulas for decades, but to date no strategy has been confirmed to be effective in 2 consecutive randomized controlled studies.

This paper focusses on evidence-based technical aspects of laparoscopic distal pancreatectomy.

## 2. Methods

A systematic literature search was performed in PubMed for studies published up to November 1, 2014. Search terms included “technical aspects,” “laparoscopy,” “pancreatectomy,” “splenectomy,” “spleen preservation,” “radical antegrade modular pancreatosplenectomy,” “fistula,” and relevant synonyms. Titles and abstracts of all articles were screened for eligibility. Subsequently, full-text English articles on the technique of laparoscopic distal pancreatectomy and prevention of POPF were included in this study. Articles with the highest methodological quality were selected for inclusion, for every topic separately.

## 3. Results

### 3.1. Laparoscopic Distal Pancreatectomy

Benign neoplasms of the distal pancreas only have to be treated in case of incapacitating symptoms. Premalignant neoplasms have to be resected to prevent the progression to cancer. For pancreatic cancer, surgery is the only potential curative therapy. Until recently, distal pancreatectomy was a relatively rare procedure, mainly due to the low incidence of pancreatic tumors and the high proportion of unresectable disease at first presentation. In last years, the total amount of distal pancreatectomies performed each year has increased and this is suspected to be mainly because of the increased use and improved quality of diagnostic imaging, causing subsequent detection of pancreatic cysts [7]. A laparoscopic approach to distal pancreatectomy is gaining popularity and is by some considered the standard approach to distal pancreatectomy for benign and premalignant disease [8]. However, this procedure can be technically demanding and should be performed only by surgeons with sufficient experience in both open pancreatic surgery and advanced laparoscopic gastrointestinal surgery [9].

Especially in the beginning of the 21st century, many cohort series on laparoscopic distal pancreatectomy appeared [10]. As a logical consequence, in the past 3 years, several systematic reviews and meta-analyses on laparoscopic versus open distal pancreatectomy have been published [1, 2, 11–17]. From these meta-analyses it can be concluded that the most obvious benefits of laparoscopic distal pancreatectomy compared with open distal pancreatectomy are a postoperative earlier recovery, a reduced length of hospital stay, and potentially a higher spleen preservation rate [1, 2, 11–16, 18]. The benefit of the laparoscopic approach regarding reduced hospital stay remains also in case-matched studies [15]. Unfortunately, randomized controlled studies have not been performed.

Only a small minority of studies on laparoscopic versus open distal pancreatectomy focused on cancer. In previous studies on open surgery for cancer, the radical antegrade modular pancreatosplenectomy (RAMPS) seemed superior to conventional distal pancreatectomy concerning radical resection margins and lymphadenectomy [19], but large cohorts of laparoscopic RAMPS procedures are lacking.

Surgeons should not be hesitant to convert a laparoscopic distal pancreatectomy or RAMPS procedure to an open procedure if patient safety including oncological efficacy is compromised. Within the current literature, conversion rates range from 0% to 33% and this may be related to the surgeon's learning curve but should not be seen as a complication [20–24]. Main reasons in the literature for conversion from laparoscopic to open distal pancreatectomy are severe bleeding, extensive tumor growth, and lack of progress [21, 23, 25].

#### 3.1.1. Trocars and Mobilization of the Pancreas

**Figure 1:**
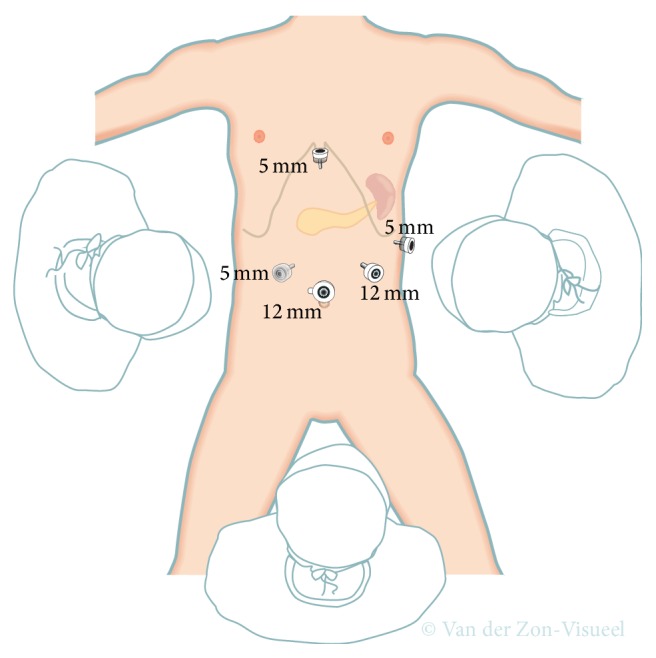
Trocar placement for laparoscopic distal pancreatectomy. Transparent 5 mm trocar is the additional trocar recommended during laparoscopic distal pancreatectomy for cancer as it will facilitate lymphadenectomy at the hepatic artery and celiac trunk.

The technique of laparoscopic distal pancreatectomy can vary widely between different surgeons and centers. Several studies reported low intraoperative blood losses, short operative times, and low conversion rates [26–30]. The technique used in these series was comparable in several aspects. Four to five trocars are placed in a semicircular fashion centered around an umbilical camera, as shown in Figure 1. The gastrocolic ligament is opened with an energy device, while preserving the gastroepiploic vessels. The stomach is lifted with a suture to permit access to the lesser sac and good exposure of the pancreas. Two large bites of a nontied suture through the posterior fundus are used, which are then led out next to the epigastric port (shown in Figure 1) [26, 27]. Alternatively, the stomach can be retracted with a laparoscopic retractor device. Intraoperative ultrasound can be performed at this stage, to define the precise location of the lesion, its relation to the splenic vessels, and the level of resection needed. Then the pancreas can be mobilized, first by mobilizing the inferior pancreatic border, which creates access to the posterior pancreatic surface. Further dissection of the superior pancreatic border permits placement of a nylon tape around the pancreas. This nylon tape can be used to delineate the surgical planes and to enable further mobilization. During laparoscopic distal pancreatectomy, the best option is to first transect the pancreas and then separate the splenic vessels from the pancreatic specimen in a medial to lateral approach using electrocautery or/and an ultrasonic/sealing device. Small vessels from the splenic artery or vein to the pancreas can be transected. This has to be performed with caution, as bleeding posterior to the pancreas may be difficult to control and necessitates occlusion of the splenic blood flow. Slinging the pancreas at both sides of the tumor with nylon tape and splenic vessels with vessel loops delineates the surgical plain and ensures a better surveillance of peripancreatic tissue. The pancreas and splenic vessels can be easily secluded from surrounding structures using this technique, which simplifies pancreatic transection without damaging main vessels [28–30]. Slinging the splenic vessels with vessel loops is facilitated by using a laparoscopic 90-degree serrated grasper. If the lesion is located distally in the tail, a lateral to medial dissection can be attempted.

#### 3.1.2. Spleen Preservation

Distal pancreatectomy with subsequent splenectomy is indicated in case of malignant disease of the distal pancreas to ensure extensive resection of lymph nodes located along the splenic artery and the splenic hilum [6, 31]. Splenectomy is also often performed because of technical reasons, such as vascular tumor involvement, but potentially because spleen preservation can be technically challenging. However, in case of nonmalignant disease, preservation of the spleen is advised, as spleen preservation was seen to be associated with a reduction of perioperative infections and length of hospital stay [5, 32–34]. The introduction of the laparoscopic approach in distal pancreatectomy has been associated with higher rates of spleen-preserving distal pancreatectomy (odds ratio of 3 for laparoscopy compared with open surgery), as shown in several systematic reviews [12–14]. Although the exact explanation is lacking, it has been suggested that the enhanced view during laparoscopic surgery with better visualization of splenic vessels has contributed to this finding.

In 1996, a technique of spleen-preserving distal pancreatectomy was described by Kimura et al. [3], including preservation of the splenic artery and vein. The authors concluded that this procedure is easy and safe. Contradictory to this statement, this spleen-preserving technique is known to be technically demanding, as the splenic vessels have to be dissected circumferentially and have to be separated from the pancreas carefully. In case of proven benign disease, this procedure is indicated. In case of premalignant disease, this procedure is indicated when the lesion is not attached to the spleen or splenic vessels. Otherwise, respectively, a subsequent splenectomy or spleen-preserving distal pancreatectomy with resection of the splenic vessels has to be performed.

In 1988, a spleen-preserving distal pancreatectomy with transection of the splenic vessels was introduced by Warshaw [4]. During this procedure, now often referred to as the Warshaw technique, the splenic artery and vein are transected at the side of the portomesenteric vein and at the splenic hilum. Vascular control can be achieved with Hem-o-lok clips (Teleflex Medical, Weck Drive, Research Triangle Park, NC, USA) or staplers [27, 35]. It is important to emphasize that two Hem-o-lok clips are needed to secure the vessels. An adequate vascular cuff should be left to preclude the clips from slipping off.

**Figure 2:**
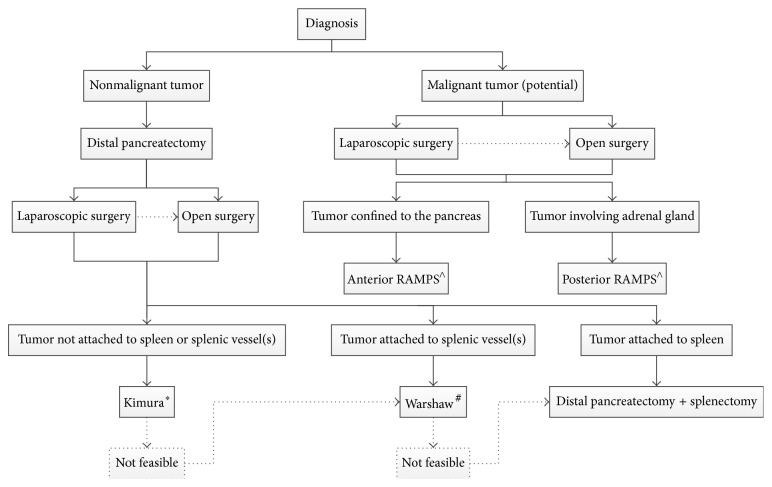
Flow chart indicating preferred surgical technique. Dotted lines are optional pathways. Laparoscopic procedures can convert to open surgery. ^*∗*^Kimura = spleen-preserving distal pancreatectomy with preservation of the splenic vessels. ^#^Warshaw = spleen-preserving distal pancreatectomy with resection of the splenic vessels. ^∧^RAMPS = radical antegrade modular pancreatosplenectomy.

**Figure 3:**
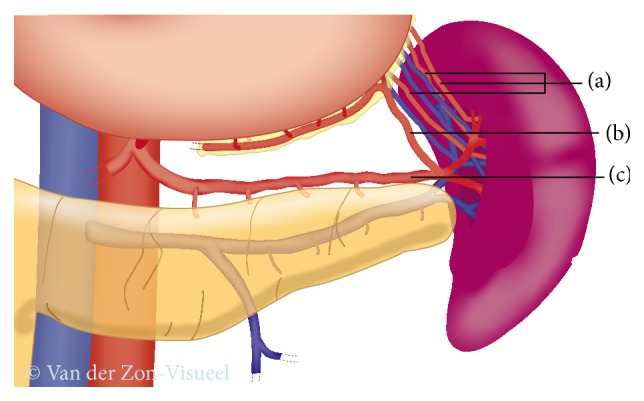
Splenic blood supply. (a) Short gastric arteries; (b) left gastroepiploic artery; (c) splenic artery.

It is unclear whether the Kimura or Warshaw technique is superior [36–38]. However, a recent systematic review reported more spleen-related complications after the Warshaw technique compared with the Kimura technique, such as need for postoperative splenectomy (2% versus 0%, *P* = 0.001), (partial) splenic infarction (22% versus 2%, *P* < 0.001), and chronic abdominal pain (38% versus 0%, *P* = 0.048) [39]. Therefore, first a Kimura technique should be attempted, with initially preserving the short gastric arteries. When this technique is not feasible, a Warshaw technique can be performed (Figure 2). In this scenario, the splenic vessels are transected. Attention should be given to preserve the left gastroepiploic artery, as this is suspected to play an important role in the prevention of postoperative splenic ischemia (Figure 3). A vascular endostapler is also a good option for vessel transection, but this may be too bulky. Again, slinging the splenic vessels with vessel loops ensures clipping and transecting these vessels easily. When a Warshaw technique is applied to preserve the spleen, the use of a bulldog arterial clamp is optional and temporarily decreases the splenic blood flow. During this procedure, alternatively the splenic artery could subsequently be dissected medially to the level of the potential pancreatic resection line before transecting the pancreas [28].

During a spleen-preserving procedure the surgeon should always examine the splenic perfusion at the end of the procedure. When signs of splenic ischemia are present, such as extensive ischemic zones, a splenectomy has to be performed. This may occur in 10% of patients [36].

#### 3.1.3. Technique for Pancreatic Cancer

**Figure 4:**
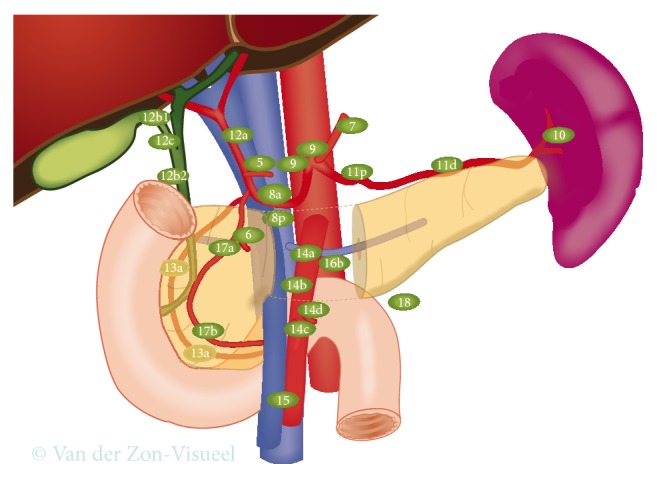
Peripancreatic lymph node stations. According to the International Study Group on Pancreatic Surgery guidelines during distal pancreatectomy for cancer lymph nodes in stations 10, 11, and 18 have to be resected. Resection of lymph nodes in stations 8a and 9 is optional, but it is suggested to be included in the resection in case of cancer located in the body of the pancreas [6].

Survival after distal pancreatectomy for pancreatic cancer is poor. To improve these outcomes, the RAMPS was developed by Strasberg et al., which is based on a medial to lateral resection, a posterior dissection plane, resection of Gerota's fascia (and optional (i.e., “modular”) the left adrenal gland), and adequate lymph node dissection [31]. Before starting RAMPS, a staging laparoscopy is indicated to rule out peritoneal or liver metastases. When the adrenal gland is not involved, an anterior RAMPS can be performed, always including resection of Gerota's fascia. Whenever the posterior margin of the tumor seems to involve the adrenal gland, a posterior RAMPS should be performed, including resection of involved organs (e.g., adrenal gland or kidney). During both the anterior and posterior RAMPS, additional lymph node dissection along the celiac trunk is performed, based on the pancreatic lymph node system as described by O'Morchoe [40]. However, a recent consensus statement of the International Study Group on Pancreatic Surgery specified that lymph node dissection of stations 8 and 9 along the common hepatic artery and the celiac trunk has to be performed only in case of malignant tumors of the body of the pancreas [6]. They stated that lymphadenectomy of station 10 (in the splenic hilum), station 11 (along the splenic artery), and station 18 (along the inferior border of the pancreatic body and tail) should be mandatory during all distal pancreatectomies for cancer. The statement did mention that the evidence-base is weak for these recommendations. See Figure 4 for a detailed illustration of peripancreatic lymph node stations.

A laparoscopic approach to the RAMPS is considered a technically difficult procedure, but it appears to be feasible laparoscopically [41]. The current evidence on laparoscopic RAMPS for pancreatic ductal adenocarcinoma is limited and, therefore, until now it is uncertain whether the oncological feasibility of laparoscopic and open approach is comparable. A recent systematic review by Ricci et al. included studies on laparoscopic versus open distal pancreatectomy for pancreatic cancer [42]. They found resection margins, lymph node retrieval, and survival to be comparable for both groups [42]. However, the available evidence is limited and further research is needed. For safety duties, one of the most recent series on this topic stated that patients suitable for a laparoscopic approach should meet the Yonsei criteria, defined as tumors which are confined to the pancreas, located 1-2 cm from the celiac trunk, with an intact fascial layer between the distal pancreas, the left adrenal gland, and the left kidney [41]. A flow chart is shown in Figure 2, summarizing which surgical procedure is preferred. The decision whether a laparoscopic or open approach has to be chosen needs to be made according to the surgeon's preference and may be based on several factors, such as the surgeon's experience, tumor size, involvement of other organs (adrenal, duodenum, stomach, colon, or kidney), and tumor morphology (e.g., infiltrating growth or inflammation).

During laparoscopic resection for cancer it is probably advisable to avoid more than 30-degree left tilt. More tilt may worsen the access for lymphadenectomy around the hepatic artery and celiac trunk. Placement of an additional trocar at the right of the infraumbilical camera port, as shown in Figure 1, will further facilitate this lymphadenectomy.

### 3.2. Postoperative Pancreatic Fistula

Although the mortality associated with pancreatectomy has decreased, the morbidity remains high, ranging from relatively low to very high percentages [43, 44]. The most frequent complication after distal pancreatectomy is a postoperative pancreatic fistula, occurring in 4% to 69% of patients [45, 46]. POPFs are associated with several further complications, such as intra-abdominal abscesses, subsequent sepsis, wound infection, delayed gastric emptying, ileus, and postpancreatectomy hemorrhage.

The International Study Group on Pancreatic Fistula (ISGPF) developed a consensus definition and grading scale to aid in classifying POPFs [47]. The definition of a postoperative POPF is drain output of any volume on or after postoperative day 3 with an amylase greater than 3 times the upper normal serum level. Many factors influence POPF, including patient-related risk factors (age, sex, and body mass index), disease-related risk factors (pancreatic gland texture and pancreatic duct size), procedure-related risk factors (operative time, transection, technique, closure technique, and intraoperative blood loss), and the surgeon's experience [48–50].

Unfortunately, we can conclude that a laparoscopic approach to distal pancreatectomy failed in reducing the rate of POPF [51, 52]. Nevertheless laparoscopy is associated with better outcomes than open surgery, so new methods for the prevention of POPF should be applicable during or after laparoscopic surgery.

The ISGPF definition of POPF seems not to be ideal for laparoscopy since the length of hospital stay is reduced when compared to conventional surgery and therefore more patients will go home with a drain in situ [1, 15, 16]. According to the ISGPF definition, patients are having grade B POPF, rather than grade A, when they are discharged with a surgical drain [47]. After laparoscopy, the decision to discharge a patient is not driven by the presence of a surgical drain and many patients will be able to go home on day 3 or 4 postoperatively. Due to the definition, this could potentially somewhat increase the reported rate of grade B POPF.

Once the diagnosis of POPF is established, conservative management leads to successful outcome in about 90% of cases [53]. Depending on the patient's clinical conditions, sometimes interventional radiological assistance is required, but reoperation is very rarely indicated [48, 54]. Several resection methods and closure techniques of protecting the pancreatic remnant have been developed especially in an effort to reduce POPF after laparoscopic distal pancreatectomy. These include transection and closure using a stapling device, oversewing the staple line, pancreatic transection using various energy devices, staple line reinforcement, reinforcement of the pancreatic stump with a jejunal loop, gastric anastomosis or falciform ligament patch, sealing with fibrin sealant patches, pancreatic sphincterotomy, and administration of somatostatin analogues. Most of these techniques can be applied during laparoscopic surgery. Hereafter, we summarize the evidence of the four most important approaches to the prevention of POPF.

#### 3.2.1. Preventing Fistula 1: Perioperative Endoscopic Intervention

Perioperative endoscopic pancreatic sphincterectomy has been proposed to prevent POPF, mainly after distal pancreatectomy [55, 56]. This technique is highly feasible and is usually well tolerated by patients [57]. Recent randomized controlled trials showed that prophylactic pancreatic stenting does not reduce POPF after distal pancreatectomy [58]. In fact, it increased bacterial seeding in the stent, leading to formation of abscesses. The ongoing randomized controlled trials in Sweden and the United States will hopefully be able to show the true value of this technique [59, 60].

#### 3.2.2. Preventing Fistula 2: Transection and Closure Techniques

Several techniques for transecting the pancreas and treating the pancreatic remnant after distal pancreatectomy have been reported [61–68]. Until now, only Suzuki et al. concluded from the first randomized controlled trial that the use of ultrasonic dissectors significantly reduced the incidence of POPF [69].

Hand-sewn closure and stapler closure represent the most common techniques of pancreatic remnant management and can both be performed during laparoscopic distal pancreatectomy. The evidence showed that stapler closure is not superior to hand-sewn closure [46, 70]. Recently, the DISPACT trial showed that incidence of POPF after hand-sewn closure was not reduced compared with stapler closure, as it was 28% versus 32%, respectively (*P* = 0.56) [71]. This outcome is quite relevant, as stapler closure is the preferred technique during laparoscopic distal pancreatectomy. The results obtained with stapler closure might be improved by advances in stapling devices. For instance dividing the pancreas using 2.5 mm vascular staple cartridges significantly decreased the rate of clinically significant pancreatic fistula compared with 4.5 mm staple cartridges [72]. Furthermore, gradual closing of the stapler over the course of about 2 to 3 minutes could reduce POPF rates as reported by Asbun and Stauffer [73]. They reported a cohort of laparoscopic distal pancreatectomies and applied this stepwise stapler closing method, which resulted in an acceptable grade B/C POPF rate of 6.6% [73].

In recent years some evidence on the effect of reinforcement of a stapled transection line on POPF appeared. In 2008, an American research group published promising results, as they found that staple line reinforcement after distal pancreatectomy significantly reduced the POPF rate from 25% to 10% (*P* < 0.02) [74]. A few years later, this was confirmed in a randomized controlled trial from Hamilton et al. [75]. They randomized 100 patients to either mesh (*n* = 54) or no mesh (*n* = 46) reinforcement of the pancreatic transection line and found ISGPF grade B/C POPF to be reduced significantly from 20% to 1% in case of a mesh (*P* = 0.0007).

#### 3.2.3. Preventing Fistula 3: Glue and Patches

During laparoscopic distal pancreatectomy some other techniques of POPF prevention could be utilized, such as fibrin glue and sealant patches [76–82]. However, the results of retrospective reviews and trials are contrary and, therefore, the use of these techniques is the subject of further research and discussion. There have been reports using gastric and jejunal patches, covering the pancreatic stump. Moriura et al. reported that pancreas related complications decreased when using a seromuscular patch [83]. However, these results were not confirmed in the randomized controlled trial by Oláh et al., in which grade B/C POPFs after stapler transection and stapler transection combined with a seromuscular patch were found to be similar [84]. Additionally, the application of a falciform ligament patch and fibrin glue to standard stapled or sutured remnant closure did not reduce the rate of POPF in patients undergoing distal pancreatectomy [85]. In 2012, Montorsi et al. [82] published the results of a randomized controlled trial on the effect of an absorbable fibrin sealant patch on POPF after distal pancreatectomy. In total, 275 patients were enrolled, of which 145 were allocated to the absorbable fibrin sealant patch group and 130 to the control group. This study showed comparable POPF rates in both groups, both for total POPF rates and grade B/C POPF rates [82]. To date, it is unclear whether these kinds of interventions will decrease POPF rates significantly after laparoscopic distal pancreatectomy or not.

#### 3.2.4. Preventing Fistula 4: Somatostatin Analogues

Somatostatin is an inhibitor of endocrine and exocrine pancreatic activity. The use of somatostatin analogues for preventing POPF is controversial [86]. However, Gurusamy et al. conducted a Cochrane analysis and showed reduction of complications and POPF rate using somatostatin analogues, without decreasing clinical relevant POPF rates [87]. The efficacy of prophylactic somatostatin analogues was reported to be improved, by selective administration in the setting of high-risk patients, including patients with a soft pancreatic gland, a small pancreatic duct or patients in whom intraoperative blood loss was excessive [88]. Since evidence on the benefit of its use is still lacking, administration based on the surgeon's interpretation of the risk of POPF at the time of surgery is required to establish clear guidelines [89]. Interestingly, the newest results from a trial by Allen et al. [90] showed that the use of pasireotide (a long half-life somatostatin analogue) in the perioperative period significantly reduced risk of clinically relevant POPF after distal pancreatectomy [90].

## 4. Conclusion

The laparoscopic approach to distal pancreatectomy is suggested to be associated with improved time to recovery and improved rates of spleen-preserving distal pancreatectomy, but randomized studies are still lacking. Several randomized controlled trials have reported effective techniques to prevent postoperative pancreatic fistula (seromuscular jejunal patch, mesh reinforcement, and pasireotide injection), but no strategy has been confirmed in a second consecutive randomized controlled trial. Further research on the prevention of POPF is warranted.
